# Tissue-Specific Transcriptomes Reveal Gene Expression Trajectories in Two Maturing Skin Epithelial Layers in Zebrafish Embryos

**DOI:** 10.1534/g3.119.400402

**Published:** 2019-08-20

**Authors:** Shawn J. Cokus, Maricruz De La Torre, Eric F. Medina, Jeffrey P. Rasmussen, Joselyn Ramirez-Gutierrez, Alvaro Sagasti, Fang Wang

**Affiliations:** * Department of Molecular, Cell, and Developmental Biology, University of California Los Angeles and; † Department of Biology, California State University, Dominguez Hills

**Keywords:** zebrafish, skin development, epithelia, transcriptome, keratin

## Abstract

Epithelial cells are the building blocks of many organs, including skin. The vertebrate skin initially consists of two epithelial layers, the outer periderm and inner basal cell layers, which have distinct properties, functions, and fates. The embryonic periderm ultimately disappears during development, whereas basal cells proliferate to form the mature, stratified epidermis. Although much is known about mechanisms of homeostasis in mature skin, relatively little is known about the two cell types in pre-stratification skin. To define the similarities and distinctions between periderm and basal skin epithelial cells, we purified them from zebrafish at early development stages and deeply profiled their gene expression. These analyses identified groups of genes whose tissue enrichment changed at each stage, defining gene flow dynamics of maturing vertebrate epithelia. At each of 52 and 72 hr post-fertilization (hpf), more than 60% of genes enriched in skin cells were similarly expressed in both layers, indicating that they were common epithelial genes, but many others were enriched in one layer or the other. Both expected and novel genes were enriched in periderm and basal cell layers. Genes encoding extracellular matrix, junctional, cytoskeletal, and signaling proteins were prominent among those distinguishing the two epithelial cell types. *In situ* hybridization and BAC transgenes confirmed our expression data and provided new tools to study zebrafish skin. Collectively, these data provide a resource for studying common and distinguishing features of maturing epithelia.

Epithelial cells form sheets that surround and define vertebrate organs. All epithelial cells share certain features, including apical–basal polarity, adhesion via cell–cell junctions, and expression of keratin intermediate filaments that provide mechanical strength. Notwithstanding these similarities, specific epithelial cell types have distinguishing features: they can form a single layer or stratify into a multi-layered epithelium; they can be flat (squamous) or tall (columnar); they can project a variety of elaborate protrusions, such as microvilli, cilia, and stereocilia; they can associate with a basement membrane on their basal surface or specialized extracellular matrices (ECMs) on their apical surfaces; and they can form different types of junctions. Here we set out to define the gene expression programs that determine common and distinguishing features of distinct epithelial cell types as they mature.

Much is known about the stem cells and complex regulatory mechanisms that maintain the stratified epidermis of the adult vertebrate skin during homeostasis and wound healing (Gonzales and Fuchs 2017), but relatively less attention has focused on the embryonic skin, before stratification. The vertebrate embryonic skin is initially a bilayered epithelium, consisting of an outer periderm and an inner basal cell layer (Wolf 1967, 1968a, 1968b; Holbrook and Odland 1975, 1980; Herken and Schultz-Ehrenburg 1981; M’Boneko and Merker 1988; Le Guellec *et al.* 2004; O’Brien *et al.* 2012; Richardson *et al.* 2014). In zebrafish, periderm cells are specified early in development from the enveloping layer surrounding gastrulating embryos (Kimmel *et al.* 1990), and differentiate a few hours ahead of basal cells (O’Brien *et al.* 2012). In mammals, periderm differentiates from surface ectoderm in a stereotyped regional progression (Wolf 1967, 1968a; Herken and Schultz-Ehrenburg 1981; M’Boneko and Merker 1988; Hardman *et al.* 1999; Richardson *et al.* 2014). Once specified, basal and periderm cells independently proliferate (Herken and Schultz-Ehrenburg 1981; Lee *et al.* 2014). Basal cells are stem cells that eventually give rise to all keratinocytes of the stratified adult epidermis (Smart 1970; Fuchs and Raghavan 2002; Muroyama and Lechler 2012; Guzman *et al.* 2013; Lee *et al.* 2014). Periderm in both fish and mammals is a transient tissue that ultimately sloughs off later in development (Wolf 1967, 1968a, 1968b; Holbrook and Odland 1980; M’Boneko and Merker 1988; Lee *et al.* 2014; Richardson *et al.* 2014).

Periderm and basal cells illustrate the key differences distinguishing epithelial cell types. For example, basal cells secrete a basement membrane on their basal surfaces to separate the epidermis from the mesenchymal dermis (Fuchs and Raghavan 2002; Muroyama and Lechler 2012), whereas periderm cells lack a basal ECM but display a glyocalyx and associate with mucins on their apical surfaces (Wolf 1967; Pinto *et al.* 2019; Depasquale 2018). Periderm cells project actin-based projections on their apical surfaces, either microvilli or elongated structures called microridges (Wolf 1967, 1968a; Lam *et al.* 2015; Depasquale 2018). Befitting their function as the embryo’s main barrier to the external environment, periderm cells are connected to one another by tight junctions (Morita *et al.* 2002; O’Brien *et al.* 2012; Yoshida *et al.* 2012; Richardson *et al.* 2014), whereas basal cells lack tight junctions, but have hemidesmosomes that attach them to the basement membrane (Le Guellec *et al.* 2004; Li *et al.* 2011a; O’Brien *et al.* 2012; Muroyama and Lechler 2012). Zebrafish basal cells have a unique interaction with the touch-sensing axon endings that innervate the zebrafish skin: initially, sensory axons innervate the region between the two cell layers, but, by 54 hr post-fertilization (hpf), the apical membranes of basal cells have begun wrapping around axons to ensheath them in structures reminiscent of sheaths formed by non-myelinating Schwann cells (O’Brien *et al.* 2012; Jiang *et al.* 2019). Since zebrafish are externally fertilized, and transgenic reporters for basal cells and periderm cells are available, zebrafish embryos are an ideal model for studying these early events in epithelial development.

As a foundation for understanding the maturation of epithelial cells and, more specifically, the function and development of basal and periderm cells, we determined the expression profiles of each cell layer at several early developmental timepoints. To accomplish this, we purified each cell type from transgenic zebrafish with Fluorescence-Activated Cell Sorting (FACS), and deeply profiled gene expression in each isolated population with RNA-Seq. These data identify genes that may be responsible for common and distinct features of periderm and basal cells, and provide a reference for studies of epithelial cell maturation.

## Materials and Methods

### Zebrafish

Zebrafish were maintained in 28.5° and pH 7.5 fish water. Adults were kept in a 14-hour light / 10-hour dark cycle. Tg(*krt4:dsRed*), Tg(*krt5:GFP*), Tg(*krt5:Gal4*), Tg(*UAS:nfsB-mCherry*), and Tg(*ΔNp63:Gal4*) lines were previously described (Curado *et al.* 2007; Hu *et al.* 2010; O’Brien *et al.* 2012; Rasmussen *et al.* 2015). Animal care and experimental procedures were approved by the CSUDH IACUC Committee and the UCLA Chancellor’s Animal Research Committee.

### Fluorescence-Activated Cell Sorting (FACS)

Dechorionated embryos were transferred into 1.5 ml tubes and rinsed with Ca^2+^-free Ringer’s solution for 15 min. During this incubation, yolk was removed by gently pipetting embryos through a 200 µl tip (with the end cut off) three to five times. Yolk-free embryos were transferred into a 35 mm petri dish with 5 ml of 0.25% Trypsin-EDTA (Sigma-Aldrich). Embryos were incubated at 28.5° and homogenized with a 200 µl tip every 10 min until most cells were dissociated (20 to 50 min). 55 µl 100 mM CaCl_2_ and 550 µl FCS were added to stop digestion. Dissociated cells were transferred into a 15 ml tube and centrifuged at 300 g for 5 min at 15°. Cells were rinsed once with 10 ml suspension solution (colorless Leibovitz medium L-15 with 0.3 g/L glutamine, 0.8 mM CaCl_2_, Penicillin 50 U/ml, Streptomycin 0.05 mg/ml, and 1% FCS). Dissociated cells were resuspended in suspension solution to ∼10^7^ cells/ml and immediately proceeded to cell sorting at the UCLA Broad Stem Cell Research Center Flow Cytometry Core. BD FACS ARIA II SORP instruments sorted cells, using a 488 nm laser for GFP detection and a 561 nm laser for dsRed detection.

### RNA-Seq libraries

Sorted cells in suspension solution were immediately lysed using the Qiagen RNeasy kit. Lysis was completed within two hours of dissociating cells. Total RNA was isolated following the Qiagen RNeasy kit and stored at –80°. Quality of all samples was assayed with an Agilent Bioanalyzer, and only samples with RNA Integrity Number > 8 were used for creating sequencing libraries. Poly-A bead-purified RNA-Seq libraries were prepared with the Illumina TruSeq RNA Library Prep Kit.

### Sequencing

23 RNA-Seq libraries (one per experimental replicate) were run a total of 25 times in various combinations across five full and fractional single end 50 or 51 nt Illumina HiSeq 2000/2500 high output lanes (with ∼1% PhiX spike-in as internal control), and demultiplexed (allowing a single mismatch to expected 7-mers) to obtain 21.2 to 45.4 million PF = 1 reads per replicate. Adapters at 3*ʹ* read ends and low quality bases were trimmed with *CutAdapt* 1.8.1 (Martin 2011, -m 21 --trim-n --max-n=2 -q 10,10 - a AGATCGGAAGAGCACACGTCTGAACTCCAGTCAC); in each replicate, ≥ 98.2% of reads and ≥ 97.9% of bases survived.

### Alignment

For cross-experiment fairness of mapping, only the first 47 nucleotides of each trimmed read were kept (retaining only trimmed reads ≥ 47 nt long). The resulting 21.0 to 45.2 million reads per replicate were aligned (with per-base quality scores) to the Ensembl release 92 (http://apr2018.archive.ensembl.org/Danio_rerio/Info/Index) top-level reference Zebrafish genome (GRCz11 chromosomes 1 to 25 + mitochondrion + 847/120-sequence subset of KN/KZ scaffolds, augmented with PhiX; alternate sequences excluded) with *STAR* 2.5.3a (Dobin *et al.* 2013, in single pass mode using known junctions from the Ensembl release 92 Zebrafish top-level reference annotation set [consisting of 31,901 genes — of which 25,431 are protein_coding — and 58,867 transcripts, of which 51,233 belong to protein_coding genes], retaining multiple locations for non-unique reads; --alignEndsType EndToEnd --outFilterMultimapNmax 999 --quantMode TranscriptomeSAM GeneCounts --outSAMtype BAM SortedByCoordinate --outSAMunmapped Within --outMultimapperOrder Random --outSAMattributes All --alignIntronMin 10 --alignIntronMax 700000 --chimSegmentMin 20 --chimSegmentReadGapMax 3 --chimMainSegmentMultNmax 999 --winAnchorDistNbins 12 --winBinNbits 17 --winFlankNbins 6). Per replicate, 96.4–97.6% of reads aligned, and, of those aligning, 85.7–95.5% aligned uniquely.

### Quantification

Per-library count distributions to the Ensembl release 92 top-level reference Zebrafish transcripts were formed with *Salmon* 0.10.2 (Patro *et al.* 2017, submitting and including a patch that became part of *Salmon* 0.11.0 to fix a bug discovered during this work) from the *STAR* transcriptome alignments (--numGibbsSamples=1000 --thinningFactor=25 --useVBOpt --libType=U --fldMean=200 --fldSD=200 --rangeFactorizationBins=4 --minAssignedFrags=1 --seqBias --noBiasLengthThreshold). For each replicate, 1,000 Gibbs bootstrap count distribution samples were retained to enable technical variance estimation in statistical analyses.

### Statistical analyses

We followed the general outline of R Bioconductor package *Sleuth *(Pimentel *et al.* 2017, as if the *Wasabi* package (https://github.com/COMBINE-lab/wasabi) was used to import) in modeling per gene, per condition log-scale expression as normal distributions, with variance partitioned into a technical, assay component (informed by *Salmon* bootstraps) and a biological component (informed by replicate experiments within conditions). However, we manually conducted all analysis steps to have greater control and incorporate some features of the *DESeq2* package (Love *et al.* 2014) that *Sleuth* does not support (*e.g.*, we use *p*-values with 1.5x-fold change thresholds, rather than for any detectable difference); details are in supplemental methods (File S1).

### Gene ranking for Figure 5

This analysis focused on genes that (1) were highly enriched in skin, and (2) had strong layer-specific expression. Using normalized transformed per-condition model mean counts (see File S1), we scored (1) as *A* – *N*, where *A* was the mean of the three all skin conditions, and *N* was the mean of the three nonskin_1_ conditions; and we scored (2) as the sum-of-absolute-deviations-from-mean for the four basal cell and periderm conditions. Genes were then ordered by descending sum of these two component scores.

### In situ hybridization

*In situ* hybridization was performed according to the Thisse Lab *In Situ* Hybridization Protocol 2010 Update (updated from: Thisse and Thisse 2008). The 1,073 bp *keratin 4* (*krt4*) probe was synthesized using primers 5′-TAAGACCCTCAACAACCGCT-3′ and 5′-TAATACGACTCACTATAGGGCTACCGTATCCTGACCCACC-3′. The 1,122 bp *aerolysin-like protein 1* (*aep1*) probe was created using primers 5′-TGGGTTTGGGTTGGAGGATG-3′ and 5′-CATTAACCCTCACTAAAGGGAAGCGTGTGAGTGTGTGTATGC-3′. The 617 bp *transcobalamin beta b* (*tcnbb*) probe was generated using primers 5′-GCACTGGGAGGACTGGTAAG-3′ and 5′-TTGGAGTATTACAATGCTGGAGA-3′. The 1,265 bp *hephaestin-like 1a* (*hephl1*) probe was constructed using primers 5′-GGACATCAGCATGCAGAGAA-3′ and 5′-CCCAGCAAATACCACTTCGT-3′. For each gene, 100 ng of probe was added into 400 µl of hybridization mix and hybridization incubation was conducted overnight at 70°.

### BAC transgenes

We generated translational fusion transgenes by inserting a GFP reporter gene cassette directly preceding the stop codon of target genes in Bacterial Artificial Chromosomes (BACs) (Suster *et al.* 2011). To create pCS2+_linkEGFP_KanR, the amino acid linker sequence 5′-GGGSGGG-3′ was added upstream of the EGFP initiation codon by PCR amplification and the resulting linkEGFP fusion gene was cloned in place of *gal4FF* in plasmid pCS2+_gal4FF_kanR (Bussmann and Schulte-Merker 2011) using the restriction enzymes BamHI and XbaI. To create TgBAC(*col28a1a*-EGFP), the linkEGFP_KanR cassette was recombined in place of the *col28a1a* stop codon in BAC CH211-174D12 using the primers 5′-GCAACCGCTTTGAAACAGAGGACATTTGTAAAAGCACTTGTGTGCAGACAGGATCCGGTGGAGGGT-3′ and 5′-GCTTGATAAAAAACACAATCTGCTGAATGATGCTTCATTGTCAGAGTGTGTCAGAAGAACTCGTCAAGAAGGCG-3′. To generate TgBAC(*oclnb*-EGFP), primers 5′-CAAAACTCTCCCTCATCAAAAGAAGGGTTAGCGACTACGACCACAGACAA-3′ and 5′-GTCCAATTGTAAAACCAACACGTATGCCTTGCTGAGTTTCCAGCGCCAGG-3′ were used to recombine the linkEGFP_Kan cassette in place of the *oclnb* stop codon in BACs CH73-37J7 and CH211-65N9. BAC transgenes were isolated with a Qiagen Midi kit and microinjected into embryos generated by two sets of transgenic lines, Tg(*krt5:Gal4*) crossed with Tg(*UAS:nfsB-mCherry*), and TgBAC(*ΔNp63:Gal4*) crossed with Tg(*UAS:nfsB-mCherry*) (Curado *et al.* 2007; Rasmussen *et al.* 2015) to create transient GFP expression in skin cells.

### Microscopy

For confocal imaging, live zebrafish embryos were mounted in 0.02% tricaine and 1% low melt agarose, and imaged with a Zeiss LSM 710 or 800 confocal microscope. For *in situ* hybridization images, fixed and stained embryos were placed in 100% glycerol and imaged with a Leica M165 FC Stereomicroscope, with attached Leica DMC2900 camera.

### Comparison to de la Garza

See File S1 for methodological details, including conversion of 85% of the pre-2009 Ensembl transcript identifiers involved to current genes. The modernized periderm profile (converted from de la Garza *et al.* 2013 Supplemental Table S1) containing 1,151 genes (all but five of which are protein coding), *dnIrf6*-inhibited profile (converted from de la Garza *et al.* 2013 Supplemental Table S2) containing 344 genes (all protein coding), and the intersection of these containing 87 genes were compared to our study (see File S1 and File S7).

### Data availability

Fish lines and BAC transgenes are available upon request. Reads, quantifications, and statistical intermediates and results are available within NCBI GEO series GSE132304. File S1 contains supplemental methods. File S2 contains comprehensive heatmaps of Gene Ontology enrichments (retaining all rows and columns containing any *p*-value ≤ 0.0001), extending Figure 4. File S3 contains a comparison of keratin genes and chromosomal locations between our findings and Krushna Padhi *et al.* 2006. File S4 contains InterPro domain hit summaries used in the compilation of zebrafish type I and II keratins. File S5 contains comprehensive heatmaps of expressed genes (those with normalized transformed [log_2_-scale] counts ≥ 2.5 [*i.e.*, ≥ ∼5.7 in linear scale] in at least one condition) that encode cell surface receptors, transcription factors, and actin-binding proteins, extending Figure 5B–D. For convenience, File S6 repeats our gene flow classifications included inside NCBI GEO GSE132304. File S7 contains details of the comparison between our study and de la Garza *et al.* (2013). Figure S1 contains a phylogenetic tree of keratin and intermediate filament genes in zebrafish. Figure S2 contains gene expression plots (in similar style as Figure 2A) for all type I and II keratin zebrafish genes. A searchable website with gene expression profiles plotted in the style of Figure 2A is available at https://zfishskin.net. Supplemental material available at FigShare: https://doi.org/10.25387/g3.8266763.

## Results

### Developmental transcriptomes for two skin epithelial layers

To identify the genetic programs that drive maturation of periderm and basal cells, and to determine their distinguishing characteristics, we profiled gene expression at three developmental stages: the 20 somite stage (20 SS occurs at approximately 19 hpf in embryos raised at 28.5° (Kimmel *et al.* 1995), when the two epithelial skin layers are not yet fully defined; 52 hpf, when the two epithelial layers are established; and 72 hpf, when both layers have matured. These timepoints encompass the development of key epithelial features, including the formation of cell–cell junctions, the production of a specialized extracellular matrix, microridge morphogenesis on periderm cells, and formation of axon sheaths by basal cells (O’Brien *et al.* 2012).

**Figure 1:**
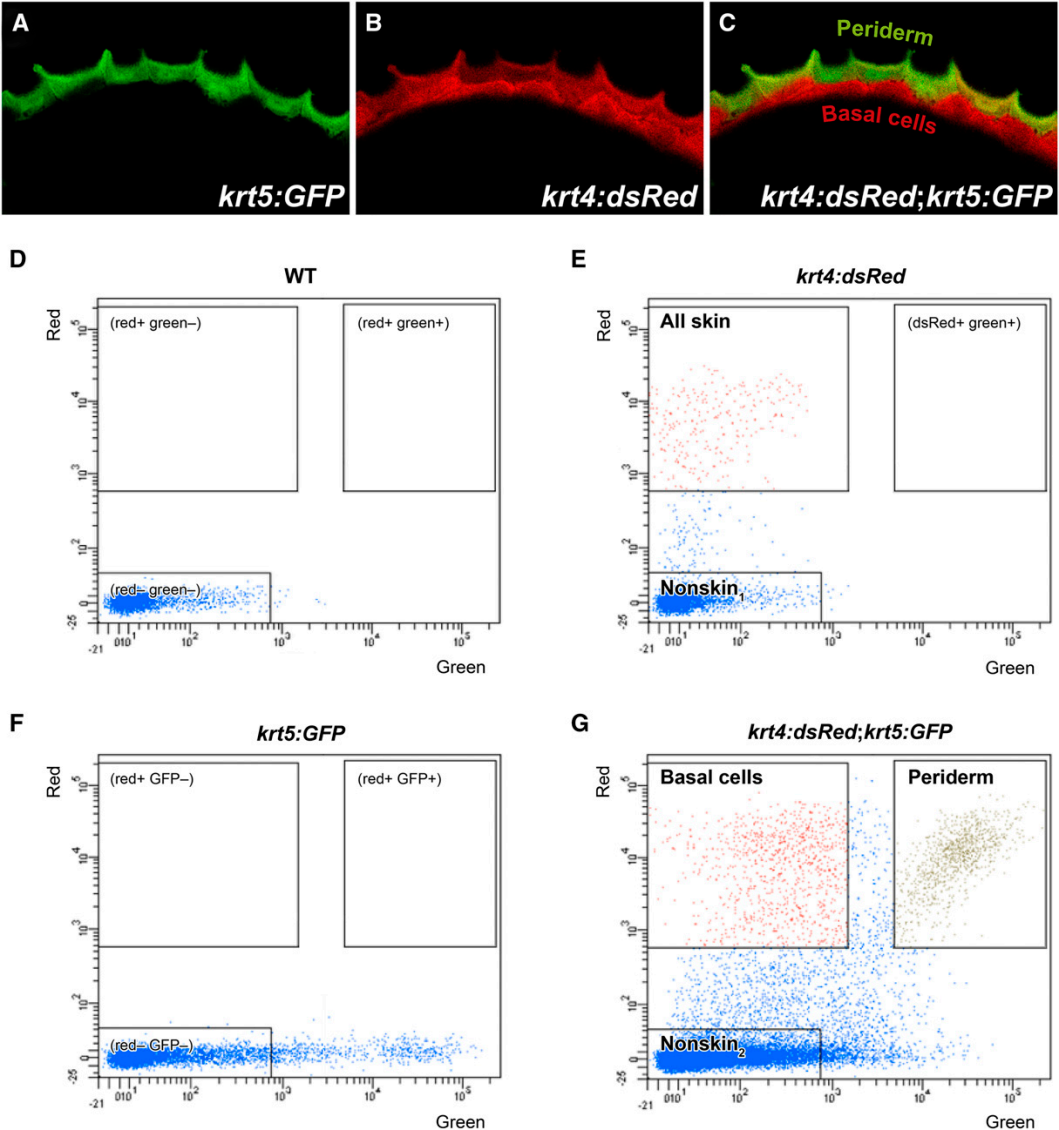
Two epithelial skin cell types isolated with FACS from transgenic fish. A–C) Confocal images of the skin covering the dorsal head region in 52 hpf embryos expressing GFP in periderm (Tg(*krt5:GFP*)), and RFP in both skin layers (Tg(*krt4:dsRed*)). D–G) Cells dissociated from 52 hpf embryos were separated by FACS. For each panel, green and red fluorescence intensities are indicated on *x*- and *y*-axes, respectively. FACS plots show gates for each cell population from wildtype animals (D), Tg(*krt4:dsRed*) embryos (E), Tg(*krt5:GFP*) embryos (F), and embryos with both transgenes (G). Cells in “All skin”, “Nonskin_1_”, “Basal cells”, “Periderm”, and “Nonskin_2_” boxes show gates that were used to collect cells into separate tubes for generating RNA-Seq samples.

To isolate each cell type, we used two transgenic fish lines: *krt4:DsRed* and *krt5:GFP* (Hu *et al.* 2010; O’Brien *et al.* 2012). Expression of the *krt4:DsRed* reporter begins at an early embryonic stage and labels both skin cell layers, but not other tissues, at all three developmental stages (O’Brien *et al.* 2012; Wang *et al.* 2012). *krt5:GFP* is not significantly expressed at 20 SS, but exclusively labels periderm cells (and not basal cells) at 52 hpf and 72 hpf (Hu *et al.* 2010). By crossing these two lines, we obtained double transgenic embryos that enabled the separation of skin cells (expressing *krt4:DsRed*) from the rest of the embryo (no fluorescence) at 20 SS. At 52 hpf and 72 hpf, these transgenic reporters distinguished periderm cells (expressing both transgenes) from basal cells (expressing only *krt4:DsRed*) as well as the rest of the embryo (no fluorescence) (Figure 1A–C).

Using FACS, we separated dissociated cells into specific isolates (Figure 1D–G). This enabled construction of the following 12 experimental conditions: “all skin cells” (RFP+) at 20 SS, 52 hpf, and 72 hpf; “nonskin_1_” cells (RFP–) at 20 SS, 52 hpf, and 72 hpf; “periderm” (RFP+ GFP+) at 52 hpf and 72 hpf; “basal cells” (RFP+ GFP–) at 52 hpf and 72 hpf; and “nonskin_2_” cells (RFP– GFP–) at 52 hpf and 72 hpf. We isolated two biological replicates for each condition, except for a single iteration of nonskin_2_ cells at 52 hpf.

An RNA-Seq library was prepared, sequenced, aligned, and quantified for each replicate experiment to obtain per-experiment, per-transcript estimated read count distributions (Methods and File S1); genes were handled as arithmetic sums of their transcript isoforms. The Gibbs samples of quantifications afforded estimates of per-gene technical variance, and biological replicates within conditions in combination with a shrinkage procedure enabled estimation of per-gene biological variance. Normalized expected read counts for genes (averaged over replicates within conditions), with awareness of total (technical plus biological) variance for each gene, were used for statistical analyses (*e.g.*, for differential expression).

**Figure 2:**
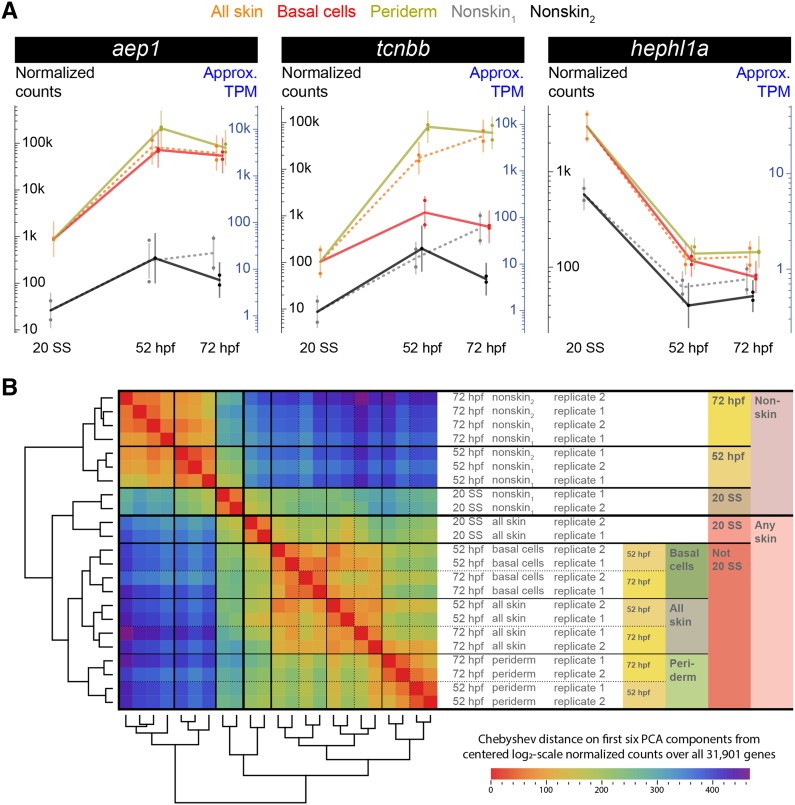
RNA-Seq transcriptomes reveal gene expression profiles, and cluster analysis of experiments. A) Three example expression profiles. In each plot, 23 dots (one for each experiment) show normalized expected read counts; dot color and placement indicates condition (with slight horizontal jitter to reduce visual overlap). For each condition, a vertical bar indicates the statistical model normal distribution (see File S1), with the bar vertically centered at the mean, and with tips at twice standard error away from the mean (hence, approximately indicating 95% confidence intervals). Approximate Transcripts Per Million (TPMs) are also shown (see File S1). Lines connect tissue means across timepoints (at 20 SS: using nonskin_1_ for nonskin_2_, and all skin for periderm and basal cells). B) Blind clustering of experiments: centered unscaled Principal Components Analysis (PCA) was performed on normalized transformed (log_2_-scale) expected counts for all 23 experiments using all 31,901 genes. The distance matrix and dendrograms after hierarchical clustering on the first six PCA components (using complete linkage with Chebyshev distance and optimal swiveling to minimize sum of adjacent leaf distances) are shown. The largest difference was between nonskin and skin conditions. Among nonskin experiments, timepoint was the next largest difference. In skin, 20 SS *vs.* 52/72 hpf was the second largest difference, followed by layers, and finally 52 hpf *vs.* 72 hpf. At 52 and 72 hpf, experiments involving all skin cells were more similar to basal cells than to periderm.

To visualize each gene’s expression pattern, we plotted its expression across time per tissue. For example, Figure 2A shows three previously unknown skin enriched genes: *aep1*, *tcnbb*, and *hephl1a*. (These three genes were selected for verification by *in situ* hybridization (Figure 6).) At 52 hpf and 72 hpf, *aep1* was highly expressed in both layers, whereas *tcnbb* was specifically enriched in periderm cells. By contrast, *hephl1a* was expressed at much higher levels at 20 SS than at later stages. Similar plots for all genes, searchable by Ensembl identifier or gene names/keywords, are available on a website (https://zfishskin.net).

### Dynamics of gene expression in epithelial skin layers during early development

For a high level overview of the relationships among the genetic programs of different cell types, we performed blind clustering of the experimental expression profiles (Figure 2B). Replicate-to-replicate variation was smaller than cross-condition variation, except among nonskin_1_ and nonskin_2_ experiments, which were quite similar. As expected, the transcriptomes of skin *vs.* nonskin populations were most different from one another. Cell populations were then divided by timepoint: for both skin and nonskin, profiles at the 20 SS stage were clearly more different from those at the 52 and 72 hpf timepoints than the latter two stages were from each other. Skin samples next diverged in expression by layer, with 52 and 72 hpf only distinct at the finest levels of comparison (apart from replicate experiments) at this scale. At 52 and 72 hpf, the expression profiles of samples involving all skin cells were between those of basal cells and periderm, consistent with all skin being a physical mixture of the layers. These observations illustrate that, despite their differences, distinct epithelial cell types share much in common.

To study the different dynamic patterns of gene expression, we classified every gene into categories at each timepoint (see File S1 and File S6). At 20 SS (when we did not separate the two skin layers), each gene expressed significantly more highly in all skin relative to nonskin_1_ was placed into the “skin genes” (S) category, and the rest into the “nonskin genes” (N) category. At each of 52 and 72 hpf, a gene was placed into one of four categories based on the probabilities of the various orderings that periderm, basal cells, and nonskin_2_ cells could have had by the gene’s expression at that timepoint, with a 1.5x-fold change threshold for significant differences. A non-negligible probability (≥ 0.02) that neither periderm nor basal cells was significantly higher than nonskin_2_ cells placed the gene in the “nonskin” (N) category; otherwise, a non-negligible probability that periderm and basal cells were not significantly different placed the gene in the “general skin” (G) category; and otherwise the gene was placed in the “periderm-preferred” (P) or “basal-preferred” (B) categories, according to which layer was more likely highest. Placing genes into these categories allowed us to identify both common and distinguishing features of periderm and basal cells. Altogether, 23% of all 31,901 genes — the 7,286 “skin-enriched genes” — were in a skin category (S/G/B/P) for at least one timepoint. Although our libraries were constructed with poly-A purification and our focus was on protein-coding genes, 10% of skin-enriched genes were annotated as non-protein coding (compared to 20% of all genes), with some detected at considerable levels.

**Figure 3:**
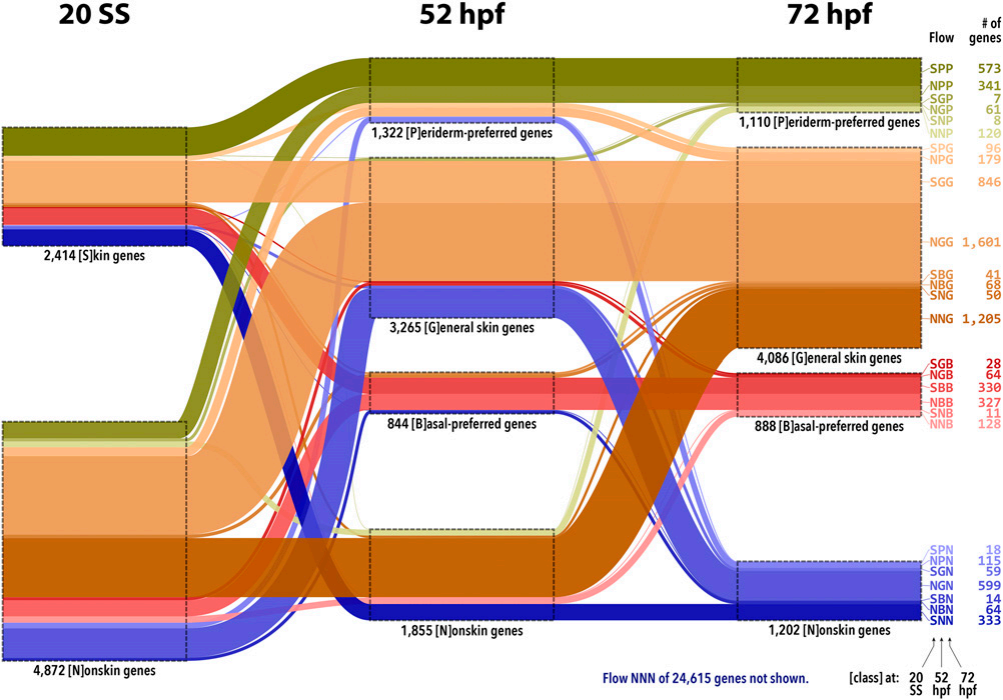
Expression dynamics of skin-enriched genes. Using RNA-Seq expression profiles, the 31,901 Ensembl release 92 zebrafish genes were classified (see File S1) at 20 SS into categories of ‘S’kin genes *vs.* ‘N’onskin genes, and at each of 52 and 72 hpf into categories of ‘P’eriderm-preferred genes, ‘B’asal-preferred genes, ‘G’eneral skin genes, and ‘N’onskin genes. Each gene thus belongs to exactly one of 32 possible “flows”, of which 28 have at least one gene (all of which except NNN are depicted, with width proportional to the number of genes). 15 flows have at least 70 genes, and 10 flows have at least 200 genes.

Visualizing expression dynamics of skin-enriched genes revealed basic features of the skin developmental program. We refer to a combination of categories at 20 SS, 52 hpf, and 72 hpf from the previous paragraph as a gene expression “flow”, and abbreviate such combinations by concatenating the category characters for 20 SS, 52 hpf, and 72 hpf, in that order. Figure 3 represents all flows for the 7,286 skin-enriched genes. For example, 573 genes were enriched in skin at 20 SS (category S) and later became specifically enriched in periderm cells (category P) at both 52 and 72 hpf; this flow is thus denoted “SPP”. At 20 SS, there were 2,414 skin genes, while there were 5,431 genes and 6,084 genes in G/B/P categories at 52 and 72 hpf, respectively, indicating that major aspects of cellular specification occurred between 20 SS and 52 hpf, and fewer between 52 hpf and 72 hpf. The largest changes between 52 and 72 hpf were exchanges between general skin genes and nonskin genes, but more genes changed from nonskin to general skin (∼2:1), suggesting that subsequent maturation likely involves recruitment of new genes.

Basal- and periderm-preferred genes were quite stable. The four flows involving B *vs.* P exchanges — SBP, SPB, NBP, and NPB — had *zero* genes. Thus, of the 844 basal-preferred genes at 52 hpf, zero were periderm-preferred at 72 hpf while 78% remained basal-preferred (and 13% became general skin and 9% nonskin, with some fraction of these expected to be borderline cases). Similarly, of the 1,322 periderm-preferred genes at 52 hpf, zero were basal-preferred at 72 hpf while 69% remained periderm-preferred (and 21% became general skin and 10% nonskin, again with some fraction expected to be borderline cases). These results suggest that fate specification for the periderm and basal cell layers is largely irreversible by 52 hpf.

### Differential functional enrichments in two distinct epithelial skin layers during early development

**Figure 4:**
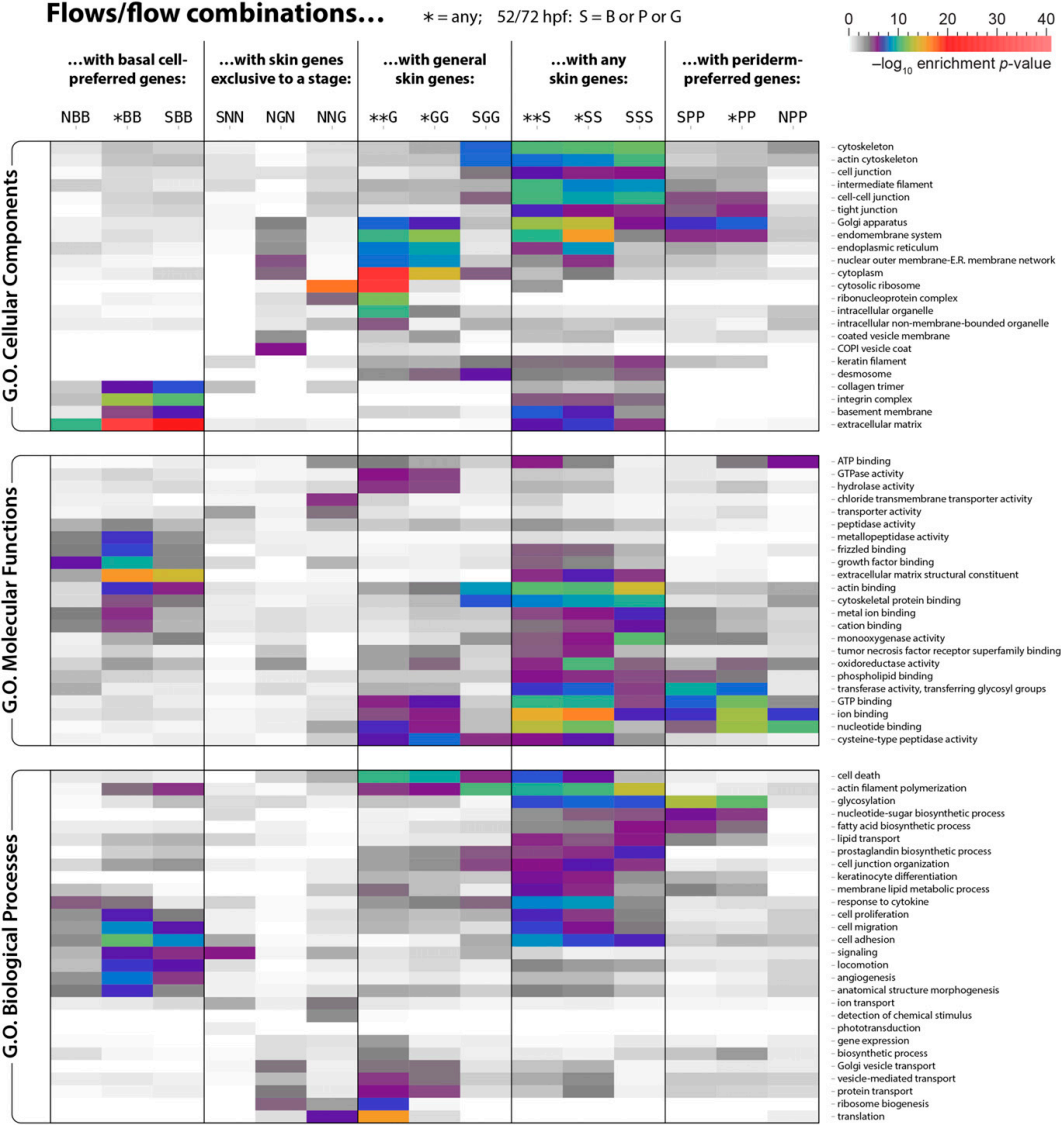
Highlights of Gene Ontology (GO) enrichments in flows and certain flow combinations. We examined GO Cellular Component, Molecular Function, and Biological Process terms for enrichment in flows (expression patterns of category ‘N’/‘S’ at 20 SS and ‘N’/‘G’/‘B’/‘P’ at 52 and 72 hpf) and certain combinations of flows (at 20 SS, ‘*’ combines ‘N’ and ‘S’; at 52/72 hpf, ‘S’ combines ‘B’ and ‘P’ and ‘G’, and ‘*’ combines ‘S’ and ‘N’), as described in File S1. The *p*-values (see colorbar; grays are insignificant) for selected illustrative GO terms and flows/flow combinations are shown. (For larger heatmaps including all rows and columns containing every *p*-value ≤ 0.0001, see File S2.)

To unveil the unique characteristics of different developmental stages and epithelial layers, we used functional annotation enrichment analyses to examine every gene expression flow, as well as certain combinations of flows. An asterisk at a timepoint indicates any expression category; for instance, “*BB” refers to genes that were in any category at 20 SS (N or S), but were in the basal-preferred category at both 52 and 72 hpf. Furthermore, at each of 52 hpf and 72 hpf, genes enriched in skin in any way were partitioned across three categories (B/P/G), and ‘S’ (“any skin”) indicates any of these three ways. For example, “SSS” indicates genes that were skin genes at 20 SS and also belonged to any of the skin categories (B/P/G) at both 52 and 72 hpf (and not necessarily the same category at 52 hpf *vs.* 72 hpf). To display some of the key biological differences distinguishing cell populations, we show 15 representative flows/flow combinations and 74 representative Gene Ontology (GO) terms — 23 Cellular Component (CC) terms, 23 Molecular Function (MF) terms, and 28 Biological Process (BP) terms — in Figure 4. (Comprehensive heatmaps are available in File S2.) These analyses revealed layer-specific gene categories, and gene categories that changed as skin matured.

The basal cell-preferred (NBB, *BB, and SBB) and periderm-preferred (SPP, *PP, and NPP) gene flows/combinations were differentially enriched in several CC, MF, and BP GO terms. Basal flows/combinations were enriched with GO terms indicating extracellular matrix (especially basement membrane) and integrin complex components; actin binding and growth factor binding functions; and cell adhesion, migration, and signaling processes. By contrast, periderm gene flows/combinations were enriched in cell–cell junction (especially tight junction) and endomembrane/Golgi components; ion, nucleotide, GTP, and ATP binding functions; and glycosylation, nucleotide-sugar, and fatty acid biosynthetic processes. Layer-specific gene flows committing to skin later (NBB and NPP) were enriched in fewer GO terms than flows for layer-specific genes committing to skin early (SBB and SPP), suggesting that later-enriched genes might be more diverse, resulting in fewer significant GO term enrichments.

Flows/combinations focusing on general skin genes (**G, *GG, and SGG) were enriched in GO terms that were mostly distinct from those for flows/combinations involving basal cell-preferred or periderm-preferred genes (although a few terms were shared with periderm), reflecting common skin cell features. For example, desmosome and cytoskeletal (actin) components, and actin filament polymerization, cell death, translation, and protein transport processes were enriched in flows/combinations focused on general skin, and thus represent common epithelial features. Flows/combinations involving any skin categories (**S, *SS, and SSS) more or less combined enrichments seen in the various more specific flows/combinations, as might be expected.

GO terms enriched at different stages revealed aspects of the maturation process. For example, genes that were skin genes at 20 SS, but were nonskin genes later (SNN) were enriched in signaling processes. These signaling processes could regulate steps in differentiation, or, since the peripheral axons of somatosensory neurons have just begun arborizing between the two skin epithelial layers at 20 SS (O’Brien *et al.* 2012), they might reflect interactions between skin cells and somatosensory neurons. The transient general skin gene flow (NGN) was enriched with COPI vesicle coat components, suggesting that the production of secreted or membrane proteins might be enhanced at a specific step of skin cell maturation. Chloride transmembrane transporter activity, cytosolic ribosome components, and translation were enriched in the late general skin gene flow (NNG), indicating that relatively mature epithelial cells increase active protein production.

### Characterization of keratin gene expression patterns in two skin epithelial layers

**Figure 5:**
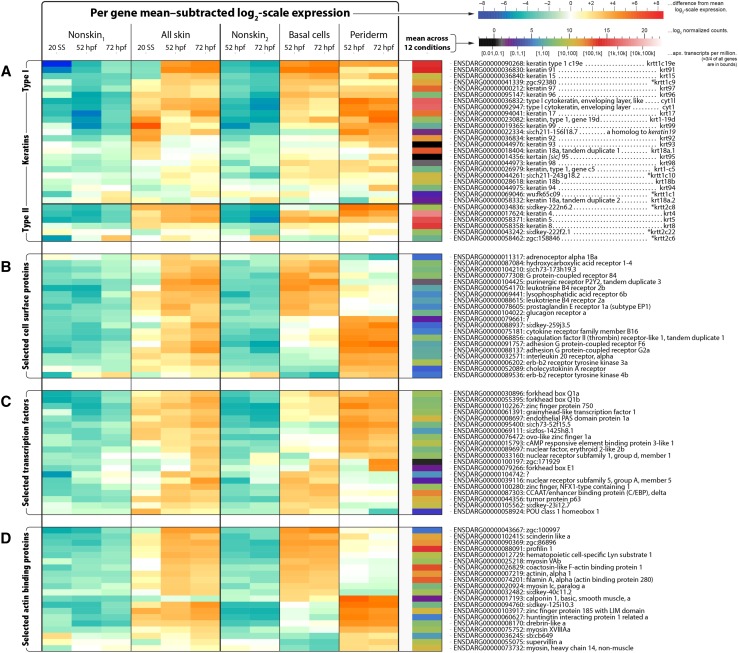
Expression for four gene classes. For A) all keratins (type I and type II), B) the top 20 cell surface receptors, C) the top 20 transcription factors, and D) the top 20 actin binding proteins, a heatmap illustrating expression across conditions is shown. For each gene, its normalized transformed (log_2_-scale) counts are gathered for the 12 conditions; the mean of these is displayed in the column to the right (see lower colorbar). The values after subtracting the mean (in linear scale, these then indicate fold changes relative to the geometric mean) are displayed to the left (see upper colorbar). For determination of genes in each class, see File S1; for the rank order used to select the top 20 genes in the cell surface receptors, transcription factors, and actin binding proteins, see Methods. Asterisks in (A) indicate previously-used gene symbols (Krushna Padhi *et al.* 2006).

Keratins are types of intermediate filaments expressed in vertebrate epithelial cells, providing them with structural integrity (Bragulla and Homberger 2009; Coulombe and Lee 2012). Although keratin filaments are abundant in all epithelial cells, specific keratin genes have restricted expression patterns, and are thus often used as diagnostic markers for specific cell types and developmental stages (Byrne *et al.* 1994; Conrad *et al.* 1998; McGowan and Coulombe 1998; Mazzalupo and Coulombe 2001; Lu *et al.* 2005; Krushna Padhi *et al.* 2006). Intracellular keratins are formed from heterodimers of type I (acidic) and type II (neutral-basic) keratin proteins. The last comprehensive analysis of keratin genes in zebrafish was reported in 2006, and identified 16 type I and seven type II zebrafish keratin genes (Krushna Padhi *et al.* 2006). Since the zebrafish reference genome has significantly improved since 2006, we re-surveyed its modern gene models to identify type I and type II keratins (see File S1, File S3, Figure S1, and File S4), adding seven type I keratin genes and removing one type II gene relative to the previous analysis, resulting in a total of 23 type I and six type II zebrafish keratin genes (Figure 5A). As previously noted, many of the type I genes are clustered on chromosomes 11 and 19, and five of the new type I genes added are located in these clusters. The revised keratin gene identifications provide an updated reference for the zebrafish community.

The seven added type I genes were *krt17*, *krt18a.2*, *krt94*, *krt96*, *krt98*, *cyt1l*, and *si:ch211-156l18.7* (the last being homologous to *keratin19* in many organisms); the ones retained were *krt15*, *krt18a.1*, *krt18b*, *krt91*, *krt92*, *krt93*, *krt95*, *krt97*, *krt99*, *krt1-c5*, *krt1-19d*, *krtt1c1*, *krtt1c9*, *krtt1c10*, *krtt1c19e*, and *cyt1*. The type II genes were *krt4*, *krt5*, *krt8*, *krtt2c6*, *krtt2c8*, and *krtt2c22*; we removed *si:dkey-183i3.5* a.k.a. *krtt2c21* (Krushna Padhi *et al.* 2006) a.k.a. *thread keratin alpha* (Schaffeld and Schultess 2006). All of our accepted type II genes are much closer to one another in sequence similarity than any of them are to *si:dkey-183i3.5*, and the same holds for all of our accepted type I genes, and the InterPro domain structure details of *si:dkey-183i3.5* are not like those of our accepted type I or type II keratin genes (Figure S1 and File S4). Similar remarks apply to another gene, *zgc:136930*, also called *thread keratin gamma* (Schaffeld and Schultess 2006), and a third gene, *krt222*. However, these three genes do appear to code for intermediate filaments (File S4).

Six type I keratin genes (*krt18a.2*, *krt93*, *krt95*, *krt98*, *krtt1c1*, and *si:ch211-156l18.7*) had very low expression levels (mean expression level < ∼10 normalized counts) in all conditions, suggesting that they do not participate in early development (Figure 5A and Figure S2). The other 17 type I keratin genes and all six type II keratin genes were expressed at higher levels, and enriched at some developmental stage in at least one type of skin cell, compared to nonskin cells (Figure 5A and Figure S2). Nine type I keratin genes (*krt17*, *krt18a.1*, *krt91*, *krt92*, *krt97*, *krtt1c9*, *krtt1c19e*, *cyt1*, and *cyt1l*) were enriched in skin and highly expressed (mean expression level > ∼4,000 normalized counts; in every condition, this was at least the most extreme 3% of genes). Among these nine genes, *krt91* and *krtt1c9* were basal-preferred genes, whereas *krt17*, *krt92*, *cyt1*, and *cyt1l* were periderm-preferred genes. Furthermore, *krt91* and *krtt1c19e* were expressed at much higher levels at the two later developmental stages, whereas *krt18a.1 *and *krt92* were expressed at higher levels at 20 SS.

Among the six type II genes, *krt4*, *krt5*, and *krt8* were, by far, the most highly expressed in skin, and *krt4* was *the* gene — in every condition involving skin — with highest normalized counts of any gene of any kind. (The gene with highest normalized counts in nonskin_1_ at 20 SS was an eukaryotic translation elongation factor, and in either nonskin at 52/72 hpf were embryonic hemoglobins.) *krtt2c8* was strongly periderm-preferred, and the only layer-specific type II gene. *krt4*, *krt5*, and *krt8* were comparably highly expressed in both layers. Interestingly, the highly periderm-specific fluorescent reporter Tg(*krt5:GFP*) we used for periderm FACS purification was based on an enhancer fragment from the *krt5* gene (Hu *et al.* 2010), indicating that the elements in this fragment were not sufficient to drive expression in the native gene’s full pattern.

### Skin transcriptomes reveal genes that likely promote cell type-specific characteristics

As cells differentiate and mature, they integrate signals from their environment, activate unique transcriptional programs, and undergo morphogenetic cell shape changes driven by the actin cytoskeleton. To identify specific genes that may provide insight into how periderm and basal cells adopt distinct developmental trajectories, we examined the expression patterns of genes annotated as cell surface receptors, transcription factors, and actin-binding proteins (as defined in File S1), with an emphasis on genes that (1) were highly enriched in skin, and (2) had strong layer-specific expression. We scored genes by a sum of these two criteria (see Methods) and selected for display the top 20 genes in each category (Figure 5B–D).

Among the top 20 cell surface receptors, many are involved in G protein-coupled receptor signaling pathways (Figure 5B). For example, *G protein-coupled receptor 84*, *leukotriene B4 receptor 2a*, and *leukotriene B4 receptor 2b* were basal cell-preferred genes, while *adhesion G protein-coupled receptor F6* and *adhesion G protein-coupled receptor G2a* were periderm-preferred genes. Unsurprisingly, many of the top 20 transcription factors (Figure 5C) were expressed in a specific epithelial layer. Notably, *tp63*, a well-characterized basal cell gene (Vanbokhoven *et al.* 2011), was one of the top basal cell-preferred transcription factor genes. Although actin-related GO terms were generally enriched in skin cells of both layers (Figure 4), our gene-specific analysis (Figure 5D) revealed that each skin layer expressed distinct sets of actin-binding proteins. These genes (and other differentially expressed genes on the comprehensive lists; see File S5) are candidate entry points for investigating unique signaling pathways activated in each cell type, gene regulatory networks that specify these cells, and the cytoskeletal processes that endow epithelial cells with unique morphological features, such as microridges in periderm cells and ensheathment channels in basal cells.

### In situ hybridization and fluorescent transgenic reporters support RNA-Seq findings

**Figure 6:**
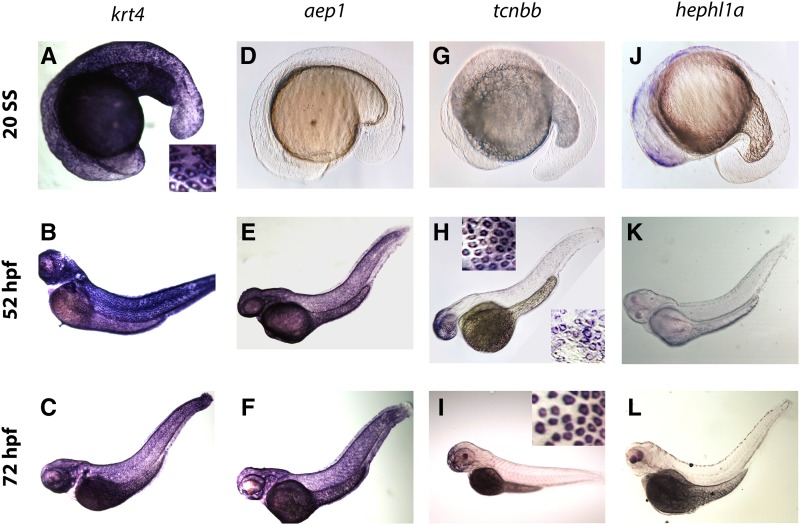
Expression patterns of four skin-enriched genes detected by *in situ* hybridization. A–C) *krt4* was highly expressed at all three stages (20 SS, 52 hpf, and 72 hpf). The inset in (A) shows an enlarged image of *krt4* expression in skin. D–F) *aep1* was only expressed highly at the two later stages (52 and 72 hpf). G–I) *tcnbb* was only highly expressed at 52 and 72 hpf. The top inset in (H) displays *tcnbb* expression in skin covering the head region, while the bottom inset shows expression in skin at the trunk region. The inset in (I) shows an enlarged image of *tcnbb* expression in skin at the anterior end. J–L) *hephl1a* expression was detected at 20 SS in skin, but not later stages. All images are oriented with dorsal to the top, ventral to the bottom, anterior to the left, and posterior to the right.

Our RNA-Seq analyses identified genes known to be expressed in skin, but also numerous genes previously unknown to be expressed in a layer- and/or developmental stage-specific manner in the embryonic skin. To confirm developmental expression patterns of representative genes, we chose novel candidates suggested by our RNA-Seq analyses and examined their expression patterns with *in situ* hybridization. Since *krt4* (Figure 6A–C) is a gene known to be strongly expressed in all skin cells at all three timepoints, it served as a positive control. The gene *aerolysin-like protein* (*aep1*) codes for a pore-forming protein, and the gene *transcobalamin beta b* (*tcnbb*) codes for a vitamin B12-binding glycoprotein; both were indicated by our RNA-Seq to be skin-enriched genes, with much higher expression levels at 52 and 72 hpf than 20 SS, whereas *hephaestin-like 1a* (*hephl1a*) codes for a metal transporter and was more highly expressed at 20 SS than 52 and 72 hpf (Figure 2A). There were limited prior expression data, primarily based on RT-PCR, for *aep1*, *hephl1a*, or *tcnbb* (Lam *et al.* 2008; Nakajima *et al.* 2011; Long *et al.* 2015; Chen *et al.* 2018; Benoit *et al.* 2018). None of these three genes had previously been suggested to be expressed in skin during early development.

We detected an *in situ* hybridization signal for *hephl1a* exclusively at 20 SS (Figure 6J–L); by contrast, *aep1* and *tcnbb* transcripts were detected in skin cells at 52 hpf and 72 hpf, but not at 20 SS (Figure 6D–I), consistent with our RNA-Seq analyses. Interestingly, a recent study indicated that Aep1 is an innate immune molecule that inhibits bacterial infection (Chen *et al.* 2018). Our results are consistent with these findings and suggest that the skin is important in innate immune responses, especially during early developmental stages. *In situ* hybridization revealed that *tcnbb* had an intriguing spatiotemporal distribution pattern, which was not possible to resolve with our RNA-Seq design: *in situ* signal was higher in skin covering the head and yolk than in other areas at 52 hpf (Figure 6H), and further concentrated in the anterior end at 72 hpf (Figure 6I). A recent study showed that *tcnbb* expression is further restricted to a unique ventral-anterior area at 5 days post-fertilization (Benoit *et al.* 2018). These observations demonstrate that our RNA-Seq analyses were sensitive enough to detect regionally restricted genes, and suggest that the skin is regionalized early in development.

**Figure 7:**
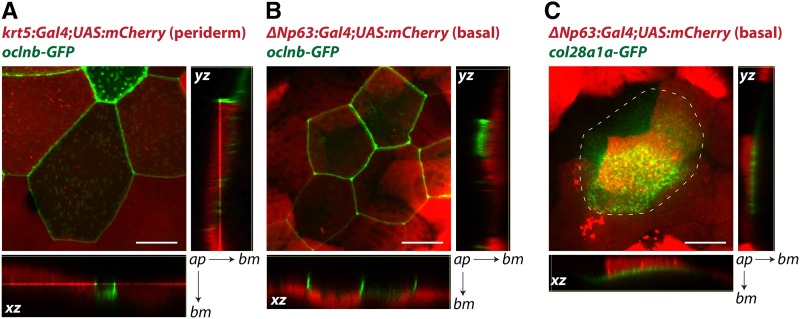
BAC transgenes illustrate layer-specific gene expression and protein subcellular distribution. A) Confocal images of a 72 hpf Tg(*krt5:Gal4*;*UAS:mCherry*) embryo with TgBAC(*oclnb*-EGFP) expression. Oclnb-EGFP localized to cell junctions in periderm cells, which also expressed mCherry. B) Confocal images of a 72 hpf Tg(*ΔNp63:Gal4*;*UAS:mCherry*) embryo with TgBAC(*oclnb*-EGFP) expression. Oclnb-EGFP was not detected in mCherry-expressing basal cells. C) Confocal images of a 96 hpf Tg(*ΔNp63:Gal4*;*UAS:mCherry*) embryo with TgBAC(*col28a1a*-EGFP) expression. Col28a1a-EGFP was detected in the basement membrane, directly basal to mCherry-expressing basal cells. Dotted line outlines area containing Col28a1a-GFP, which had spread beyond a single basal cell. Large images are 2-D (*xy*) projections from 3-D confocal *z*-stacks. Arrows point from apical surface (*ap*) to basement membrane (*bm*) for *xz*- and *yz*-planes. Scale bars are 20 µm long.

To verify layer-specific expression patterns identified by our RNA-Seq analyses, we constructed BAC transgenes with translational GFP fusions. (The estimates of absolute expression that RNA-Seq provides were especially useful for these experiments, since the utility of BAC transgenes depends on enhancer strength.) We chose the periderm-preferred gene *occludin b* (*oclnb*) and basal cell-preferred gene *collagen XXVIII alpha 1a* (*col28a1a*), as these were both strongly layer-specific and highly expressed (https://zfishskin.net). We used two sets of transgenic lines, Tg(*krt5:Gal4*) crossed with Tg(*UAS:nfsB-mCherry*) (fluorescing only in periderm), and TgBAC(*ΔNp63:Gal4*) crossed with Tg(*UAS:nfsB-mCherry*) (fluorescing only in basal cells) to separately label the two skin epithelial layers (Curado *et al.* 2007; Rasmussen *et al.* 2015). Confocal microscopy of these transgenic fish microinjected with the *oclnb*-GFP BAC transgene revealed that *oclnb* was indeed highly expressed exclusively in periderm cells and localized to cell boundaries (Figure 7A–B), consistent with its role as a tight junction component. By contrast, Collagen XXVIII alpha 1a is a member of the collagen family (Gebauer *et al.* 2016) that has not been fully characterized. Confocal images of embryos injected with the *col28a1a*-GFP BAC transgene confirmed strong GFP signal just below basal cells (Figure 7C). Strikingly, the area of GFP signal was broader than the clones of cells expressing the transgene, suggesting that Collagen XXVIII alpha 1a was secreted by basal cells and diffused within the plane of the basement membrane. Collagen XXVIII alpha 1a had not previously been reported to be a basement membrane component, demonstrating that our data has the potential to reveal novel aspects of epithelial biology.

## Discussion

By combining transgenic fish lines, cell purification, and RNA-Seq, we report comprehensive transcriptomes for two epithelial skin layers at three early developmental stages in zebrafish. Our observations verified the expression patterns of genes known to be expressed in skin, and, more importantly, identified numerous novel layer- and/or developmental stage-specific genes. This investigation can thus serve as a resource for identifying epithelial layer-specific enhancers, studying the functions of layer- and/or developmental stage-specific genes, and creating tools to label specific cell types at specific developmental stages.

Our data indicated that expression of specific junctional and ECM proteins are key cellular features distinguishing periderm and basal cells. While periderm and basal cells expressed comparable levels of adherens junction and desmosomal proteins (*e.g.*, E-cadherin and Desmoplakin a and b), only periderm cells expressed high levels of tight junction proteins, such as occludins (*e.g.*, Figure 7); and only basal cells expressed high levels of hemidesmosome proteins, such as Integrin alpha 6b (https://zfishskin.net). These expression patterns were consistent with the periderm’s role as a barrier, and the basal cell layer’s role in attachment to the basement membrane. Secreted extracellular proteins are also major distinguishing features of periderm and basal cells. Basal cells expressed high levels of basement membrane components such as Collagen IV chains (and potentially novel basement membrane-associated proteins, such as Collagen XXVIII alpha 1a), whereas periderm cells were enriched for glycosylation enzymes that likely contribute to the formation of the apical glycocalyx. Intriguingly, periderm cells also expressed high levels of at least one mucin — Mucin 13a (https://zfishskin.net). Mucins are thought to primarily be secreted by goblet cells and diffuse onto the apical surface of periderm cells (Linden *et al.* 2008). Since ECM proteins are primarily located basal to the basal cell layer or apical to periderm cells, our data could provide a useful resource for studying polarized secretion.

Our data also provide a foundation for better understanding the function of the periderm, an under-studied and relatively enigmatic tissue that is eventually sloughed off in both mammals and fish (Wolf 1967, 1968a, 1968b; M’Boneko and Merker 1988; Lee *et al.* 2014; Richardson *et al.* 2014). Periderm cells serve as the main barrier between embryos and the external environment, and thus contain tight junctions and display a glycocalyx on their apical surface. Defects in periderm differentiation or desquamation cause a variety of developmental abnormalities (Cui *et al.* 2007; Okano *et al.* 2012; de la Garza *et al.* 2013; Richardson *et al.* 2014; Liu *et al.* 2016). Our data identified specific junction genes, glycosylation factors, and transmembrane and secreted proteins that likely create these periderm-specific features. The apical surfaces of periderm cells project prominent actin-based microridges arranged in striking labyrinth-like patterns (Wolf 1967, 1968a; Lam *et al.* 2015; Pinto *et al.* 2019; Depasquale 2018). Although our study showed that actin regulatory genes as a category are enriched in both skin layers, specific actin-binding genes enriched in periderm may play specific roles in microridge morphogenesis. In mammals, the periderm disappears as the skin stratifies and the outer layers of the mature epidermis keratinize (Wolf 1968a, 1968b; Holbrook and Odland 1975; Hardman *et al.* 1999). By contrast, the basal cell-derived outer layer of the stratified adult skin does not cornify in fish, but takes on many of the characteristics of the embryonic periderm (Lee *et al.* 2014), reflecting the fact that fish skin is a mucosal epithelium at both embryonic and adult stages. Given their similarities, the periderm-like cells of the adult fish skin likely share much of the embryonic periderm gene expression program we report here.

A previous microarray-based study of the skin differentiation transcription factor Interferon regulatory factor 6 (*Irf6*) identified genes with expression enriched very early (11 hpf, before basal cells develop) in wildtype zebrafish periderm cells, as well as genes inhibited at 6 hpf by *dnIrf6*, a dominant-negative variant of *lrf6* (de la Garza *et al.* 2013). We converted the microarray periderm profile, *dnIrf6*-inhibited profile, and profile intersecting these two together to current genes to compare with our study (see Files S1 and S7). At 20 SS (our closest timepoint), all three profiles were enriched for genes we classified as ‘S’ (skin genes): 30%, 39%, and 75% of the periderm, *dnIrf6*-inhibited, and intersection profiles, respectively, were ‘S’ compared to 8% over all genes (4x to 10x higher than random expectation, hypergeometric *p*-values < 10^−53^). Expanding to all three of our timepoints, the intersection profile was strikingly enriched for our periderm classifications, with our flow SPP being the most frequent in that profile (46% of its genes: 26x, *p* < 10^−45^). In the other two profiles, SPP was the second most common flow (>7x, *p* < 10^−39^), below flow NNN. Hence, genes in these early profiles — especially the intersection profile — may indeed be key genes in establishing periderm identity.

Basal cells are stem cells that later serve as the source for stratification and diversification of the epidermis (Muroyama and Lechler 2012; Gonzales and Fuchs 2017), and are transformed in basal cell carcinomas (White and Lowry 2015). At the stages we studied, basal cells establish a unique relationship with the endings of sensory neurons that innervate the skin (O’Brien *et al.* 2012). Axons first innervate the region between basal and periderm cells, but then become enveloped exclusively by basal cells in glial-like ensheathment channels. Formation of these channels requires specific lipid microdomains, association with actin, and the formation of autotypic junctions (Jiang *et al.* 2019), but the signals that initiate ensheathment are unknown. The basal cell-specific genes we identified suggest candidate receptors for ensheathment signals, actin regulatory proteins that could promote membrane invagination, and specific components of autotypic junctions. Basal cells also play critical roles in wound healing, proliferating and migrating to repair epidermal damage. Repeating our experiments in wound paradigms (LeBert and Huttenlocher 2014) could identify basal cell genes involved in wound healing.

The cellular and molecular similarities between zebrafish and humans make them an efficient model for studying human diseases, including skin diseases (Li *et al.* 2011b; Li and Uitto 2014; Cline and Feldman 2016; Bootorabi *et al.* 2017). In recent years, larval zebrafish studies have shed light on the molecular causes and consequences of conditions characteristic of human skin diseases, including aberrant periderm development (Li *et al.* 2011a; de la Garza *et al.* 2013; Liu *et al.* 2016), defective adhesion and blistering (Carney *et al.* 2007, 2010; Sonawane *et al.* 2009; Kim *et al.* 2010; Goonesinghe *et al.* 2012; Postel *et al.* 2013), impaired wound healing (Niethammer *et al.* 2009; Rieger and Sagasti 2011; Lisse *et al.* 2016), and dysregulation of epidermal cell proliferation (Sonawane *et al.* 2005, 2009; Carney *et al.* 2007; Webb *et al.* 2008; Dodd *et al.* 2009; Eisenhoffer *et al.* 2012; Brock *et al.* 2019). Our gene expression profiles provide a resource for identifying additional molecules that may contribute to these and other skin conditions.
